# Inherited Moderate Factor X Deficiency Presenting as Cardiac Tamponade

**DOI:** 10.1155/2019/9657516

**Published:** 2019-09-25

**Authors:** Tamer Othman, Ayman Abdelkarim, Karen Huynh, An Uche, Jennifer Lee

**Affiliations:** ^1^Department of Internal Medicine, Harbor-UCLA Medical Center, Torrance, CA, USA; ^2^Division of Hematology and Medical Oncology, Harbor-UCLA Medical Center, Torrance, CA, USA

## Abstract

Factor X deficiency is a rare bleeding disorder that varies in the severity of its clinical manifestations. The symptoms of this disorder can occur at any age, although most severe cases appear in childhood. The rarity of this condition has not allowed for the establishment of evidence‐based management guidelines, and thus, individuals afflicted with factor X deficiency are treated based on limited literature and the opinions of clinicians with extensive experience. In this case report, we discuss a unique presentation of a 38-year-old male who was found to have cardiac tamponade as a result of his newly diagnosed inherited moderate factor X deficiency. This was discovered by obtaining a factor X activity assay and confirmed with genetic testing which demonstrated a missense variant on the factor X gene on chromosome 13. His management involved correction of his factor X deficiency with fresh frozen plasma, a pericardiocentesis, and placement of a pericardial window. He has been asymptomatic and without hemorrhagic episodes for the 10 months following his discharge.

## 1. Introduction

Factor X is a vitamin K-dependent coagulation factor that is produced by the liver and is responsible for converting prothrombin to thrombin [1]. Factor X deficiency can be acquired or inherited. Inherited factor X deficiency is a very rare autosomal recessive coagulation disorder that affects 1 in every 1 million people and accounts for 10% of all rare bleeding diseases [2]. Individuals with homozygous factor X deficiency generally present early in life with severe bleeding symptoms, whereas heterozygotes, though typically clinically asymptomatic, may experience major bleeding if they undergo a major surgery or traumatic injury [3]. Acquired factor X deficiency is also rare, but when it occurs it is usually in the setting of amyloidosis.

## 2. Case Summary

**Figure 1:**
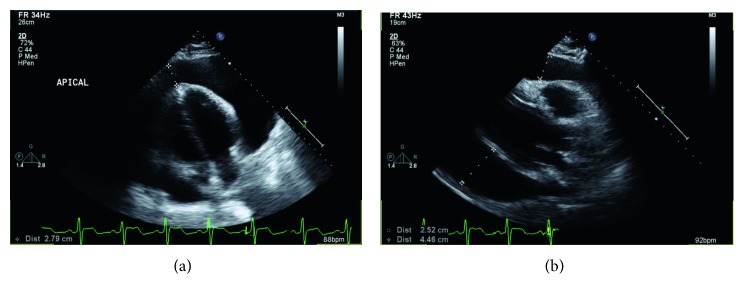
Apical (a) and parasternal (b) long axis views of the hemorrhagic pericardial effusion on presentation.

A 38-year-old Hispanic male, born in Mexico, with no known chronic medical problems presented with a 4-day history of dyspnea on exertion and orthopnea, along with positional and pleuritic chest pain. He also endorsed a dry cough that had been ongoing for months without hemoptysis. He denied fevers, chills, night sweats, unintentional weight loss, or recent trauma. He was not seen in a healthcare setting for years. He was not taking any medications at home and had no recent exposure to anticoagulants. There was no family history of bleeding disorders. He endorsed some episodes of epistaxis during the summer months and waking up in the morning with his face covered in blood in the past, but denied a personal history of bleeding from other mucosal surfaces such as gum bleeding, hematuria, hematochezia, melena, prolonged bleeding after dental procedures and a tooth extraction, swelling in joints, and spontaneous skin bruising. He had an appendectomy 14 years prior and recalls complications, such as prolonged bleeding that required blood product transfusions and a second surgery; however, details from that hospitalization are unknown. Physical exam was only remarkable for obesity, trace bilateral pretibial pitting edema, and an old appendectomy scar. Initial labs revealed a platelet count of 477 and a coagulation profile that is summarized in Table 1. A bedside ultrasound of the heart revealed a large circumferential pericardial effusion and right atrial collapse. A subsequent transthoracic echo (TTE) confirmed the large, circumferential pericardial effusion along with echo and Doppler evidence of tamponade (Figure 1). Further laboratory workup of coagulopathy revealed a factor X level of 1%. The rest of his tested factor activity levels are summarized in Table 2.

The patient received a one-time dose of aspirin 324 mg in the emergency room, and was then started on colchicine 0.6 mg twice daily for treatment of pericarditis. Nonsteroidal anti-inflammatory drugs were not administered during his hospital course due to his high risk of bleeding. The initial plan to correct his factor X deficiency in preparation for a pericardiocentesis was to administer 20 mL/kg of fresh frozen plasma (FFP) [4], but after receiving 1 unit of fresh FFP on day 4 of admission, he developed a urticarial transfusion reaction, so the transfusion was stopped and he was given famotidine 20 mg and diphenhydramine 25 mg. Due to this transfusion reaction, he was slowly administered FFP to correct his factor X deficiency over the next few days, receiving 1 more unit on day 5 and 2 more units on day 6, which he tolerated well. A pericardiocentesis with pericardial drain tube placement was performed on day 6 of admission with significant hemorrhagic drainage over the subsequent days. As a result, he received 3 more units of FFP on day 8, 4 units of FFP on day 9, and 2 units of FFP on day 10 prior to undergoing a pericardial window procedure. Postoperative TTE revealed a residual, but stable pericardial effusion without evidence of tamponade. The decision to administer FFP during his hospital course was based on his continuous hemorrhagic drainage rather than a goal factor X activity level due to his transfusion reaction and was only guided by his internationalized normal ratio (INR) prior to undergoing a procedure. He underwent reversal of his coagulopathy with oral vitamin K 10 mg for 5 days, and 13 units of FFP total. As a result, his factor X activity level rose to as high as 15%. Other options that were considered to reverse his coagulopathy were prothrombin complex concentrates (PCCs) and high-purity, plasma-derived factor X concentrate (pdFX). PCC was not administered due to a factor IX activity level above the upper limit of normal and the risk of thrombotic complications, and pdFX was not readily available at our institution. The etiology of his tamponade was thought to be related to pericarditis, with coagulopathy due to factor X deficiency leading to hemopericardium. Evaluation for amyloidosis, which included a serum protein electrophoresis (SPEP), a urine protein electrophoresis (UPEP), serum light chains, cytology of the pericardial fluid, and pathology of the surgical sample of his pericardium, was unremarkable. The patient was discharged without active bleeding or reaccumulation of the pericardial effusion. Repeat factors VII and X levels 10 months after discharge were 110% and 2%, respectively. Outpatient genetic testing revealed a homozygous missense variant that resulted in a single amino acid substitution of glycine to arginine at codon 21 in exon 1 of the factor X gene product.

## 3. Discussion

Factor X is a vitamin K-dependent serine protease that is positioned at the convergence of the extrinsic and intrinsic pathways of the clotting cascade and plays a crucial role in the coagulation pathway as the first enzyme in the common pathway to cross-linked fibrin clot formation [5, 6]. Factor X deficiency can be either inherited in an autosomal recessive manner or acquired. Acquired factor X deficiency is usually associated with amyloidosis but may also be due to anticoagulant therapy, underlying liver disease, medications (such as phenytoin), and paraproteinemia [1, 3].

The presentation of factor X deficiency is highly variable and symptoms can develop at any age. Generally, however, the more severe disorders present earlier on in life. Another case report demonstrates that moderate factor X deficiency does not necessarily present early in life or as a spontaneous hemorrhage as in our patient [7].

Patients with factor X deficiency are classified into 3 groups depending on the functional assay level: mild if it ranges from 6 to 10%, moderate if it ranges from 1 to 4%, and severe if it is <1% [1]. Our patient was diagnosed with moderate factor X deficiency as his initial factor X activity level was 1% in the acute bleeding setting and increased to 2% upon recovery 2 months later, which is likely a more accurate representation of his baseline physiological factor X activity level. His history and medical workup were negative for an acquired etiology, and genetic testing confirmed a hereditary etiology of factor X deficiency. In general, a diagnosis of inherited factor X deficiency is based upon identification of characteristic symptoms, a detailed patient and family history, a thorough clinical evaluation, and a variety of specialized tests, such as the factor X functional activity assay. Molecular genetic testing can confirm a diagnosis of inherited factor X deficiency, but is usually not necessary. In a clinical setting, molecular genetic testing can be challenging due to costs and the limited availability of the test as it is only available as a diagnostic service at specialized laboratories. Thus, the decision to pursue molecular genetic testing for factor X deficiency would be left to the discretion of the clinician.

One study looked at several important registries to summarize the bleeding symptoms reported as a result of factor X deficiency. The registries examined were the Greifswald Registry of Factor X Deficiency in Europe and Latin America, the Rare Bleeding Disorder Registry in North America, population registries of inherited bleeding disorders from the UK Hemophilia Centre Directors' Organization, and the Hemophilia Surveillance System in Iran [6]. The symptoms described included easy bruising, epistaxis, gum bleeding, menorrhagia, hematuria, hematomas, hemarthrosis, GI bleeding, intracranial hemorrhage, and umbilical cord bleeding. To our knowledge, a hemorrhagic pericardial effusion causing cardiac tamponade has not been reported as a manifestation of factor X deficiency, thus making our patient's presentation unique.

Factor X deficiency is typically suspected when both prothrombin time and activated partial thromboplastin time are elevated and correct with a mixing study. Factor X deficiency can be investigated by either a functional activity assay, an immunological assay, or a chromogenic assay. Immunologic and chromogenic assays can miss dysfunctional factor X activity and thus should not be used to screen for factor X deficiency [6].

There are currently no evidence-based management guidelines for factor X deficiency due to the rarity of this condition. Guidelines for management of factor X deficiency have been published by the United Kingdom Haemophilia Centre Doctors' Organization based on the available literature and extensive clinical experience [8]. Minor bleeding symptoms may be managed by topical therapies and antifibrinolytic agents, such as tranexamic acid or epsilon-aminocaproic acid. More severe bleeding symptoms may require factor X replacement with FFP. Alternative agents that may be used to reverse the coagulopathy from factor X deficiency include PCC and pdFX [9, 10]. Adverse events associated with FFP include allergic reactions and transfusion-related acute lung injury, while PCC is associated with hypercoagulable complications, such as disseminated intravascular coagulation (DIC) and venous thromboembolism (VTE) [6]. Investigations of the efficacy and safety of pdFX have shown no serious adverse events related to its administration [10, 11].

For patients undergoing surgery or require treatment for active bleeding, a target factor X activity level has not been established. A factor X activity level of 1–40% may be adequate for hemostasis and should not exceed 50% due to an increased risk of VTE [1, 6]. Prophylactic factor replacement therapy has been used in patients with hemophilia A and B to prevent recurrent hemarthrosis and reduce joint damage [12, 13], but there are no clear guidelines regarding prophylactic factor replacement therapy in factor X deficiency [6].

Since our patient's factor IX level was above the upper limit of normal, he was treated with FFP rather than PCC due to its association with VTE. pdFX is not readily available at our institution. FFP was given until the hemorrhagic drainage from his pericardial drain resolved. Hemostasis was achieved for this patient at a factor X activity level of 15%. Since the patient was stable with no significant bleeding, close monitoring was favored over prophylactic replacement therapy.

Of note, our patient had a factor VII level of 43% in the setting of an acute bleed. Patient's with factor VII levels >10% generally do not exhibit bleeding symptoms [14]. Therefore, the thought was that the factor X deficiency was responsible for his presentation and was thus the target of his diagnostic workup. To confirm a factor VII deficiency diagnosis however, the factor VII assay should be repeated at least once [15]. The repeat factor VII level for our patient once his bleeding resolved was 110%, which is likely a more accurate representation of his baseline physiologic factor VII activity. The mild decrease in his factor VII activity level was possibly due to consumption of his factor VII due to his ongoing bleeding on presentation, which was restored to normal limits with resolution of his bleeding.

A combined factor VII and X deficiency has been reported in the literature; however [16], this combined factor deficiency may be due to coincidental inheritance of separate coagulation factor deficiencies, such as a missense variation in both factor VII and factor X genes or a large deletion on the long arm of chromosome 13 involving both the factor VII and factor X gene. Determining the mechanism of a combined factor VII and factor X deficiency has clinical ramifications. Firstly, it can change management in certain circumstances that require coagulation factor repletion, as not all PCC preparations contain factor VII [17]. Additionally, it can assist with predicting patterns of inheritance and genetic counseling. As the patient's family denied bleeding symptoms, there are no plans for genetic testing of other family members, and as explained previously is usually not necessary to diagnose inherited factor X deficiency, but the patient was educated on the inheritance pattern of his disorder and the possibility of other family members inheriting this rare disorder. Family members may benefit from screening of coagulation studies, namely PTT, for risk stratification prior to surgical procedures.

In conclusion, factor X deficiency is a very rare disease, and as a result, not all potential bleeding complications have been reported, such as a hemorrhagic pericardial effusion causing cardiac tamponade. Furthermore, a lack of evidence-based management guidelines for this disease is due to the rarity of this condition and thus, management is based primarily on limited literature and clinical experience. This case report highlights a unique presentation of factor X deficiency and our management plan for this patient in the absence of evidence-based guidelines. Determining management by assessing clinical symptoms as opposed to laboratory values may be a valid strategy in the treatment of other rare disorders that lack evidence-based management guidelines.

## Figures and Tables

**Table 1:** Coagulation profile and mixing studies of the patient.

Prothrombin time (seconds)	69.3 (reference range: 12–14.2)
International normalized ratio	8.10 (reference range: 0.90–1.11)
Activated partial thromboplastin time (seconds)	70.5 (reference range: 24.7–35.3)
Thrombin time (seconds)	15.4 (reference range: 14.5–18.5)
PT 50/10 mixing study (seconds)	13.5 (reference range: 12–14.2)
aPTT 50/50 mixing study (seconds)	28.5 (reference range: 24.7–35.3)

**Table 2:** Factor activity assays of the patient.

Factor II activity (%)	116 (reference range: 70–150)
Factor V activity (%)	71 (reference range: 65–150)
Factor VII activity (%)	43 (reference range: 60–150)
Factor IX activity (%)	173 (reference range: 60–150)
Factor X activity (%)	1 (reference range: 60–150)
